# Yield gains and associated changes in an early yellow bi-parental maize population following genomic selection for *Striga* resistance and drought tolerance

**DOI:** 10.1186/s12870-019-1740-z

**Published:** 2019-04-05

**Authors:** B. Badu-Apraku, A. O. Talabi, M. A. B. Fakorede, Y. Fasanmade, M. Gedil, C. Magorokosho, R. Asiedu

**Affiliations:** 1 International Institute of Tropical Agriculture, P.M.B., Ibadan, 5320 Nigeria; 2 Obafemi Awolowo University, Ile-Ife, Nigeria; 3 CIMMYT- Zimbabwe, Harare, Zimbabwe

**Keywords:** Genomic selection, *Striga* resistance, Drought tolerance, Maize, Testcrosses

## Abstract

**Background:**

Maize yield potential is rarely maximized in sub-Saharan Africa (SSA) due to the devastating effects of drought stress and *Striga hermonthica* parasitism. This study was conducted to determine the gains in grain yield and associated changes in an early-maturing yellow bi-parental maize population (TZEI 17 x TZEI 11) F_3_ following genomic selection (GS) for improved grain yield, *Striga* resistance and drought tolerance. Fifty S_1_ lines were extracted from each of cycles C_0_, C_1_, C_2_ and C_3_ of the population and crossed to a tester TZEI 23 to generate 200 testcrosses. The testcrosses were evaluated under drought, artificial *Striga*-infested and optimal (free from *Striga* infestation and without limitation of water and nitrogen) environments in Nigeria, 2014-2017.

**Results:**

Gains in grain yield of 498 kg ha^− 1^ cycle^− 1^ (16.9% cycle^− 1^) and 522 kg ha^− 1^ cycle^− 1^ (12.6% cycle^− 1^) were obtained under *Striga*-infested and optimal environments, respectively. The yield gain under *Striga*-infested environments was associated with increased plant and ear heights as well as improvement in root lodging resistance, husk cover, ear aspect and *Striga* tolerance. Under optimal environments, yield gain was accompanied by increase in plant and ear heights along with improvement of husk cover and ear rot resistance. In contrast, genomic selection did not improve grain yield under drought but resulted in delayed flowering, poor pollen-silk synchrony during flowering and increased ear height. Genetic variances and heritabilities for most measured traits were not significant for the selection cycles under the research environments. Ear aspect was a major contributor to grain yield under all research environments and could serve as an indirect selection criterion for simultaneous improvement of grain yield under drought, *Striga* and optimal environments.

**Conclusion:**

This study demonstrated that genomic selection was effective for yield improvement in the bi-parental maize population under *Striga*-infested environments and resulted in concomitant yield gains under optimal environments. However, due to low genetic variability of most traits in the population, progress from further genomic selection could only be guaranteed if new sources of genes for *Striga* resistance and drought tolerance are introgressed into the population.

## Background

Maize (*Zea mays* L.) is the most important cereal crop playing a crucial food and nutrition roles in sub-Saharan Africa (SSA). It also serves as feed and industrial crop in the sub-region [1, 2]. Maize is widely cultivated in all agro-ecologies, however, the savannas of SSA provide the ideal environment for optimum expression of its yield potential due to high incoming solar radiation, low night temperature and minimized disease pressure. The availability of early and extra-early maize varieties has further facilitated the expansion of maize production into new boundaries particularly the marginal areas where annual rainfall is below 500 mm or where the soils are shallow or sandy [3]. Despite the high prospect for maize production, recurrent drought and *Striga hermonthica* parasitism are major production constraints of maize in the sub-region [1, 4, 5].

Drought has become a regular occurrence in most agro-ecologies of SSA, due to irregular rainfall patterns and climate change in the sub-region. Yield loss due to drought stress could be as high as 90% when the stress occurs from a few days to anthesis to the beginning of grain filling periods in maize [6]. *Striga hermonthica* parasitism is another major limitation to maize production, threatening the livelihood of over 300 million people in SSA [7] and accounting for an estimated loss of staple food crop valued at $7 billion yearly. Yield loss due to infestation by *Striga* vary from 0 to 100% depending on the severity of the infestation, type of variety under cultivation, climatic conditions and fertility status of the soil [8]. Farmers in the sub-region have experienced complete crop failure under severe *Striga* infestation and have often been forced to abandon their farmlands. The stresses could occur singly, but invariably, they occur simultaneously under field conditions with devastating consequences on maize production [9, 10]. Control measures for drought include planting in hydromorphic soils, application of irrigation and use of drought tolerant maize varieties while methods employed to mitigate the effect of *Striga* include hand pulling, crop rotation, use of herbicide, application of fertilizer, fallowing, trap and catch crops, seed treatments and use of *Striga* resistant maize varieties [11, 12]. However, host plant resistance/tolerance is the most effective, economical and sustainable approach to combat the combined effect of drought and *Striga* in the sub-region [13, 14].

Development of drought tolerant and/or *Striga* resistant maize populations, and improvement of such populations through phenotypic recurrent selection has proven to be an effective approach of increasing grain yield, while maintaining genetic variability within the populations [1, 15]. Use of molecular markers could fast track the breeding process when pre-flowering genotypic data are used for selection and recombination. This will ultimately lead to increased genetic gain per unit time and cost. However, the conventional marker-assisted selection method has proven ineffective because only major effect QTLs are used for selection, whereas both major and minor effect genes govern the expression of polygenic traits. Furthermore, the QTL effect estimates are not consistent across populations and environments due to epistasis (gene x gene interactions) and genotype x environment interactions. Genomic selection (GS) is an improved marker-based breeding method that could address the limitations of MAS. In the GS method, all available marker information are incorporated into a predictive model to estimate the genetic values of breeding progenies for selection [16, 17]. As such, each marker is considered a putative quantitative trait locus (QTL) for effective marker effect estimation and minimizes risk of missing small-effect QTLs [16–18]. The marker estimates are computed from training population, i.e. breeding material with both phenotypic and genome-wide marker data [19]. The marker effects are subsequently used for computation of genomic estimated breeding values (GEBVs) of new breeding lines in a population earmarked for GS. Heffner et al. [19] compared prediction accuracy of phenotypic selection (PS), conventional marker-assisted selection (MAS), and GS for 13 agronomic traits in a population of 374 winter wheat (*Triticum aestivum* L.) advanced-cycle breeding lines and found that the average prediction accuracies using GS were 28% greater than with MAS and were 95% as accurate as PS. However, such information comparing the effectiveness of PS and GS in improving maize under the prevalent contrasting biotic and abiotic stresses in SSA is very scarce.

In an effort to mitigate the combined effect of *Striga* and drought in the sub-region, the International Institute of Tropical Agriculture (IITA) developed a bi-parental maize population, TZEI 17 x TZEI 11 with combined tolerance to drought and resistance to *Striga*. The population was subjected to GS which involved one cycle of phenotypic selection and two subsequent cycles of marker-only selection for improved grain yield, drought tolerance and *Striga* resistance. This study was therefore conducted to: (i) determine the gains in grain yield obtained from GS under *Striga* infested, drought and optimal environments (ii) identify traits associated with yield gains during GS in the population (iii) estimate genetic variances and heritabilities of traits as GS progressed in the population and (iv) investigate interrelationships among measured traits in the population.

## Results

### Analyses of variance and genotype x environment interaction

Analysis of variance (ANOVA) across drought environments showed significant environment (env), cycle and entry-within-cycle effects for grain yield and most measured traits except cycle mean squares for stalk lodging and entry-within-cycle mean squares for anthesis-silking interval, root lodging, ear aspect and ears per plant (Table 1). The env x cycle interaction mean squares were significant for all measured traits while no trait showed significant effect for the interaction of entry-within-cycle with env. The env and cycle mean squares showed significant variation for grain yield and most measured traits under *Striga*-infested environments. The few exceptions included env mean square for anthesis-silking interval and ear rot and cycle mean square for ears per plant (Table 1). The entry-within-cycle effect revealed significant effects for days to anthesis and silking, plant height, stalk lodging and *Striga* damage at 8 WAP. Significant env x cycle interaction was observed for most measured traits except for ear height, stalk lodging, husk cover, ears per plant, *Striga* damage (8 WAP) and emerged *Striga* plants (8 WAP). However, no trait displayed significant effect for the interaction of entry-within-cycle with the environment. Under optimal environments, significant env, cycle and entry-within-cycle mean squares were detected for grain yield and other measured traits except cycle mean squares for root lodging and entry-within-cycle mean squares for root lodging, husk cover, ear rot, and ears per plant (Table 1). The env x cycle interaction effects were significant for most measured traits except for days to anthesis, plant height and root lodging while significant entry-within-cycle x env effects were detected for only days to anthesis and silking, anthesis-silking interval and stalk lodging.

**Table 1:** Mean squares of grain yield and other agronomic traits of testcrosses involving early-maturing yellow S_1_ families derived from four cycles of selection and tester TZEI 23, evaluated under drought, *Striga*-infested and optimal environments in Nigeria, 2014 to 2017

Sources of variation	DF	Grain yield, (kg/ha)	Days to anthesis	Days to silk	Anthesis silking interval (days)	Plant height (cm)	Ear height (cm)	Root lodging (%)	Stalk lodging (%)	Husk cover^d^	Plant aspect^e^	Ear aspect^f^	Ear rot (%)	Ears/plant	Stay green characteristic^g^	*Striga* damage (8 WAP^h^)	*Striga* damage (10 WAP)	Emerged *Striga* Plants (8 WAP)	Emerged *Striga* Plants (8 WAP)
Drought environments																			
ENV	2	1119245028^c^	15963^c^	13072^c^	161^c^	431323^c^	359854^c^	4.36^c^	246^c^	1344^c^	850^c^	914^c^	151^c^	15.442^c^	822^c^	^a^	^a^	^a^	^a^
Rep (ENV)	3	6073666^c^	12.6^c^	88.0^c^	39.8^c^	5080^c^	4138^c^	0.18	3.40	13^c^	13^c^	12.90^c^	16.07^c^	0.683^c^	8.68^c^	^a^	^a^	^a^	^a^
Block (ENV^b^Rep)	24	907820^c^	3.4	9.5^b^	3.7^b^	694^c^	189^b^	0.15^b^	2.20	1.69^c^	1.06^c^	1.10^c^	1.66^b^	0.047^c^	1.89^c^	^a^	^a^	^a^	^a^
Cycle	3	4823955^c^	105.2^c^	395.^c^1	88.0^c^	28457^c^	1303^c^5	1.31^c^	1.39	4.31^c^	7.58^c^	16.24^c^	7.19^c^	0.581^c^	72.67^c^	^a^	^a^	^a^	^a^
Entry (Cycle)	196	661562^c^	3.9^c^	7.9^c^	2.5	337^b^	158^c^	0.11	2.15^c^	0.43^c^	0.49^c^	0.53	1.60^c^	0.023	0.91^c^	^a^	^a^	^a^	^a^
ENV^b^Cycle	6	4589223^c^	46.5^c^	141.1^c^	50.8^c^	4825^c^	2240^c^	1.31^c^	3.74^b^	4.81^c^	6.63^c^	5.88^c^	6.31^c^	0.231^c^	18.92^c^	^a^	^a^	^a^	^a^
Entry (ENV^b^Cycle)	392	436,451	2.1	5.5	2.6	232	110	0.11	1.52	0.32	0.37	0.45	1.21	0.018	0.73	^a^	^a^	^a^	^a^
Error	1199	468,171	2.46	5.52	2.31	274	116	0.10	1.55	0.32	0.37	0.47	1.08	0.020	0.72	^a^	^a^	^a^	^a^
R-Square		0.91	0.96	0.91	0.67	0.88	0.93	0.62	0.65	0.94	0.91	0.90	0.67	0.81	0.86	^a^	^a^	^a^	^a^
*Striga*-infested environments																			
ENV	1	476242738^c^	1228^c^	1033^c^	0.41	42559^c^	23926^c^	92.7^c^	2207^c^	27^c^	^a^	8.61^c^	0.05	0.314^b^	^a^	231^c^	250^c^	9.77^c^	404^b^
Rep (ENV)	2	11290008^c^	33.1^c^	23.1^c^	1.96^c^	1536^c^	433^c^	34.2^c^	28.2^c^	1.90^c^	^a^	1.28^b^	2.97	0.002	^a^	5.00^c^	0.64^b^	13.33^c^	6.61^b^
Block (ENV^b^Rep)	16	1,082,715	4.9^b^	6.1	0.52	309^c^	180^c^	3.2	3.6	0.18	^a^	0.32	1.67	0.010	^a^	0.42	0.36^b^	0.70	0.67
Cycle	3	98106834^c^	35.6^c^	105.8^c^	3.80^c^	41356^c^	11045^c^	53.3^c^	14.1^c^	5.88^c^	^a^	26.06^c^	15.7^c^8	0.016	^a^	10.42^c^	7.15^c^	6.21^c^	9.41^c^
Entry (Cycle)	196	833,396	3.7^b^	4.8^b^	0.36	189^b^	75	2.5	3.8^c^	0.22	^a^	0.42	1.24	0.023	^a^	0.46^c^	0.24	0.54	0.55
ENV^b^Cycle	3	2889838^b^	9.9^b^	32.1^c^	1.85^c^	914^c^	187	13.8^c^	1.2	0.47	^a^	5.41^c^	11.24^c^	0.012	^a^	0.62	0.56^b^	2.85^c^	1.37
Entry (ENV^b^Cycle)	196	766,811	2.5	3.0	0.33	102	48	2.4	2.7	0.23	^a^	0.35	1.13	0.021	^a^	0.28	0.23	0.49	0.49
Error		912,812	2.95	3.79	0.36	150	77	2.67	2.65	0.20	^a^	0.40	1.22	0.020	_a_	0.33	0.21	0.58	0.56
R-Square		0.77	0.71	0.69	0.55	0.81	0.75	0.58	0.78	0.65	^a^	0.63	0.56	0.55	_a_	0.77	0.82	0.56	0.76
Optimal environments																			
ENV	2	107412892^c^	2278^c^	2439^c^	492^c^	146477^c^	14804^c^	3.35^c^	229^c^	3.21^c^	2.36^c^	223^c^	2878^c^	2.25^c^	^a^	^a^	^a^	^a^	^a^
Rep (ENV)	3	24896666^c^	3.6	3.0	0.07	791^c^	557^c^	0.22	17.2^c^	0.27^b^	0.49^c^	18.61^c^	0.92	0.10^c^	^a^	^a^	^a^	^a^	^a^
Block (ENV^b^Rep)	24	1,321,405	2.2	2.8	0.85	176^b^	66	0.12	1.6	0.16^b^	0.11^b^	0.40^c^	1.31	0.05^c^	^a^	^a^	^a^	^a^	^a^
Cycle	3	160011260^c^	40.9^c^	121.2^c^	23.46^c^	3954^c^9	14965^c^	0.04	12.5^c^	2.92^c^	4.41^c^	8.77^c^	18.30^c^	0.36^c^	^a^	^a^	^a^	^a^	^a^
Entry (Cycle)	196	1518340^c^	3.1^c^	3.6^c^	0.96^c^	155^c^	97^c^	0.12	1.9^b^	0.09	0.10^c^	0.26^b^	1.78	0.03	^a^	^a^	^a^	^a^	^a^
ENV^b^Cycle	6	8455631^c^	4.1	11.3^c^	3.51^c^	189	299^c^	0.04	5.6^c^	0.35^c^	0.26^c^	2.32^c^	12.46^c^	0.37^c^	^a^	^a^	^a^	^a^	^a^
Entry (ENV^b^Cycle)	392	1,068,615	2.7^c^	3.1^b^	0.89^c^	89	58	0.12	2.2^c^	0.11	0.07	0.21	1.68	0.02	^a^	^a^	^a^	^a^	^a^
Error	483	1,172,786	2.08	2.57	0.72	103	76	0.12	1.55	0.10	0.06	0.22	1.60	0.03	^a^	^a^	^a^	^a^	^a^
R-Square		0.70	0.84	0.83	0.80	0.89	0.74	0.54	0.68	0.58	0.65	0.85	0.88	0.63	^a^	^a^	^a^	^a^	^a^

^a^ − Trait not measured under the research condition; ^b^, ^c^ - Significant at 0.01 and 0.05 probability levels, respectively; ^d^Husk cover scored on a scale of 1–9, where 1 = husks tightly arranged and extended beyond the ear tip and 9 = ear tips exposed.; ^e^Plant aspect recorded on a scale of 1–9 based on plant type, where 1 = excellent and 9 = poor; ^f^Ear aspect rated on a scale of 1–9, where 1 = clean, uniform, large, and well-filled ears and 9 = ears with undesirable features; ^g^Stay green characteristic scored on a scale of 1–9, where 1 represented plants with almost all leaves green and 9 indicated plants with virtually all leaves dead; ^h^WAP = weeks after planting

**Fig. 1:**
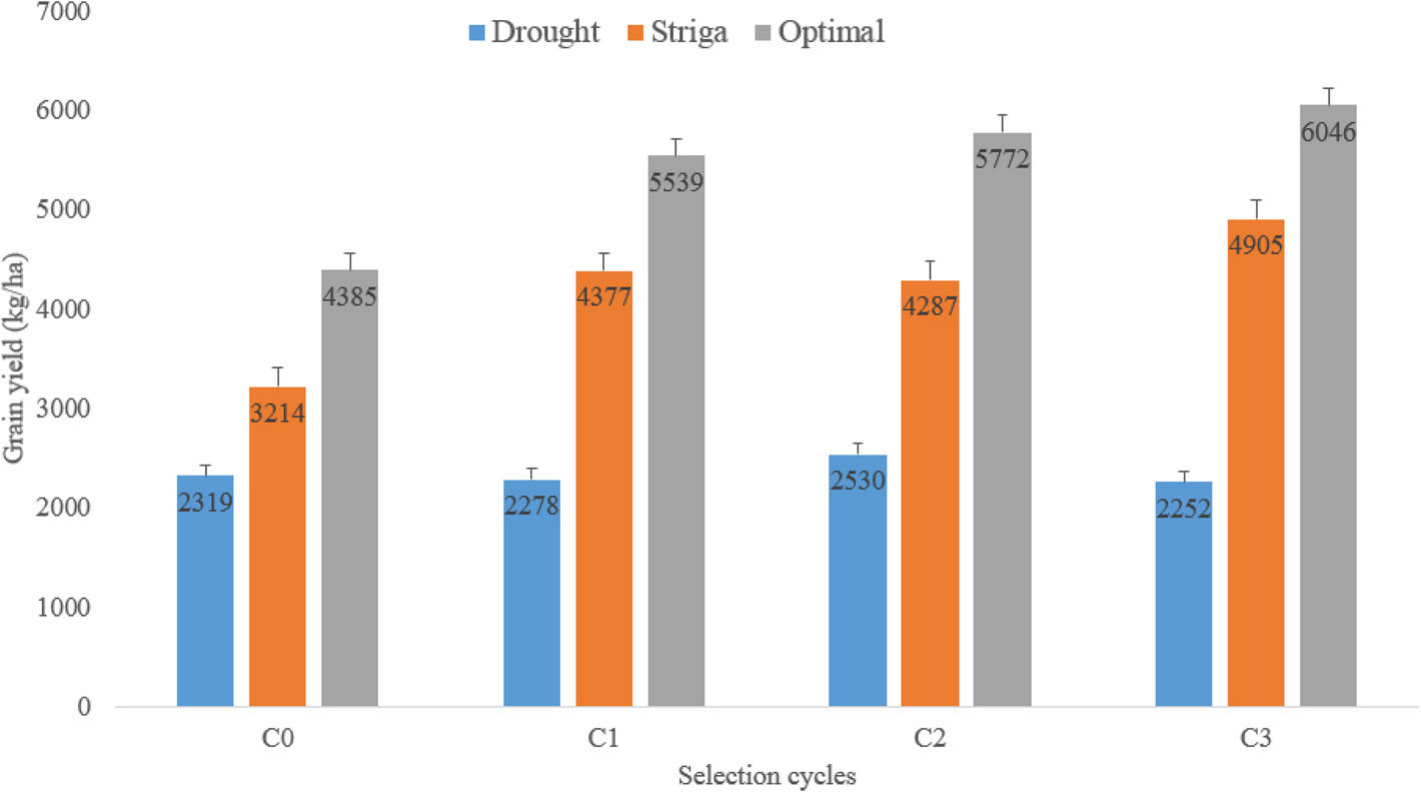
Grain yield of testcrosses involving early-maturing yellow S_1_ families derived from four cycles of selection and tester TZEI 23, evaluated under drought, *Striga* and optimal environments in Nigeria, 2014–2017

Grain yield ranged from 2252 kg ha^− 1^ for C_3_ to 2530 kg ha^− 1^ for C_2_ under drought, 3214 kg ha^− 1^ for C_0_ to 4905 kg ha^− 1^ for C_3_ under *Striga* infestation and 4385 kg ha^− 1^ for C_0_ to 6046 kg ha^− 1^ for C_3_ under optimal environments (Fig. 1). The mean grain yield of C_3_ were higher than those of C_0_ under *Striga* and optimal environments, whereas, there was no significant difference between the grain yield of C_0_ and C_3_ under drought stress.

### Gains in grain yield and changes in other traits

Significant gains in grain yield of 498 and 522 kg ha^− 1^ cycle^− 1^ corresponding to 16.9 and 12.6% gain cycle^− 1^ were detected under *Striga*-infested and optimal environments, respectively (Table 2). The yield gains under *Striga* were associated with increase in plant and ear height as well as improvement in root lodging resistance, husk cover, ear aspect and *Striga* tolerance. Gain in grain yield under optimal environments was accompanied by increased plant and ear heights along with improvement of husk cover and ear rot resistance. In contrast, genomic selection did not improve grain yield under drought stress, but resulted in delayed flowering, poor anthesis-silking synchrony during flowering and increased ear height.

**Table 2:** Relative genetic gain, coefficient of determination (R^2^), intercept (a) and regression coefficients (b) of grain yield and other agronomic traits of testcrosses involving early-maturing yellow S_1_ families derived from four cycles of selection and tester TZEI 23, evaluated under drought, *Striga*-infested and optimal environments in Nigeria, 2014 to 2017

Traits	Drought environments				*Striga*-infested environments				Optimal environments			
	Relative gain (% per cycle)	R^2^	a	b	Relative gain (% per cycle)	R^2^	a	b	Relative gain (% per cycle)	R^2^	a	b
Grain yield (kg ha^−1^)	0.22	0.0027	2332	5.1	16.9	0.82	2950	498^c^	12.63	0.85	4132	522^c^
Days to anthesis	0.83	0.9	51.5	0.43^c^	0.47	0.57	53.5	0.25	0.44	0.62	50	0.22
Days to silking	1.58	0.85	53	0.84^c^	0.85	0.64	53.9	0.46	0.71	0.52	50.7	0.36
Anthesis silking interval (days)	24.36	0.74	1.56	0.38^b^	6.59	0.24	0.91	0.06	11.53	0.27	0.85	0.098
Plant height (cm)	4.88	0.66	124.5	6.07	8.05	0.71	114.3	9.2^b^	6.45	0.77	121	7.81^b^
Ear height (cm)	5.97	0.7	72.2	4.31^b^	8.86	0.74	54.6	4.84^b^	7.52	0.74	63	4.74^b^
Root lodging (%)	−21.82	0.34	0.55	−0.12	−22.14	0.87	11.7	−2.59^c^	20	0.53	0.15	0.03
Stalk lodging (%)	0.70	0.024	4.7	0.033	−6.22	0.39	22.2	−1.38	8.84	0.18	4.75	0.42
Husk cover^d^	0.44	0.034	3.87	0.017	−3.14	0.84	3.82	−0.12^b^	−2.41	0.74	2.91	−0.07^b^
Plant aspect^e^	−0.25	0.0099	4.75	−0.012	^a^	^a^	^a^	^a^	− 2.59	0.65	3.09	− 0.08
Ear aspect^f^	0.25	0.0037	4.47	0.011	−4.50	0.79	5.56	−0.25^b^	−2.43	0.51	3.71	−0.10
Ears rot (%)	−4.92	0.21	1.22	−0.06	−9.36	0.45	1.71	−0.16	−7.86	0.87	2.29	−0.19^c^
Ears/plant	−2.74	0.43	0.84	−0.023	0.30	0.09	0.99	0.003	0.21	0.007	0.97	0.002
Stay green characteristic^g^	3.87	0.15	3.88	0.15	^a^	^a^	^a^	^a^	^a^	^a^	^a^	^a^
*Striga* damage (8 WAP^h^)	^a^	^a^	^a^	^a^	− 3.64	0.85	4.4	− 0.16^c^	^a^	^a^	^a^	^a^
*Striga* damage (10 WAP)	^a^	^a^	^a^	^a^	− 3.02	0.88	4.63	−0.14^c^	^a^	^a^	^a^	^a^
Emerged *Striga* plants (8 WAP)	^a^	^a^	^a^	^a^	12.37	0.3	5.9	0.73	^a^	^a^	^a^	^a^
Emerged *Striga* plants (10 WAP)	^a^	^a^	^a^	^a^	21.55	0.99	5.29	1.14	^a^	^a^	^a^	^a^

^a^- Trait not measured under the research condition; ^b^, ^c^ - Significant at 0.01 and 0.05 probability levels, respectively; ^d^Husk cover scored on a scale of 1–9, where 1 = husks tightly arranged and extended beyond the ear tip and 9 = ear tips exposed.; ^e^Plant aspect recorded on a scale of 1–9 based on plant type, where 1 = excellent and 9 = poor; ^f^Ear aspect rated on a scale of 1–9, where 1 = clean, uniform, large, and well-filled ears and 9 = ears with undesirable features; ^g^Stay green characteristic scored on a scale of 1–9, where 1 represented plants with almost all leaves green and 9 indicated plants with virtually all leaves dead; ^h^WAP = weeks after planting

### Genetic variances and heritability estimates

Under drought stress, genetic variance estimate was significant for one trait each in cycles C_1_ and C_3_ (Table 3). However, four to five traits showed significant genetic variances in cycles C_0_ and C_2_. The genetic variance estimates for anthesis-silking interval, stalk and root lodging, husk cover, ear aspect, ear rot and stay green characteristic were not significantly different from zero in all the selection cycles. Significant heritability estimates ranged from 0.42 for plant aspect to 0.60 for plant height in C_0_ and from 0.42 for plant height to 0.65 for days to silking in cycle C_2_. Only days to anthesis showed significant heritability estimate of 0.49 in C_1_ while ears per plant had a significant heritability estimate of 0.47 in C_3_.

**Table 3:** Estimates of genetic variance (±SE) and broad-sense heritability (±SE), of measured traits of testcrosses involving early-maturing yellow S_1_ families derived from four cycles of selection and tester TZEI 23, evaluated under three drought stress environments in Nigeria, 2014–2017

Trait	Genetic variances				Broad-sense heritability			
	C_0_	C_1_	C_2_	C_3_	C_0_	C_1_	C_2_	C_3_
Grain yield (kg ha^− 1^)	30,537 ± 18,526	23,998 ± 15,356	65,233 ± 27488^a^	22,740 ± 13,476	0.36 ± 0.22	0.34 ± 0.22	0.50 ± 0.21^a^	0.37 ± 0.22
Days to anthesis	0.30 ± 0.11^a^	0.21 ± 0.09^a^	0.37 ± 0.15^a^	0.23 ± 0.13	0.56 ± 0.21^a^	0.49 ± 0.21^a^	0.52 ± 0.21^a^	0.39 ± 0.22
Days to silking	0.17 ± 0.16	0.18 ± 0.19	0.89 ± 0.28^b^	0.41 ± 0.23	0.23 ± 0.23	0.21 ± 0.23	0.65 ± 0.20^b^	0.39 ± 0.22
Anthesis-silking interval (days)	^c^	^c^	0.14 ± 0.083	0.07 ± 0.102	^c^	^c^	0.36 ± 0.22	0.15 ± 0.23
Plant height (cm)	25 ± 9^a^	16 ± 8	16 ± 8^a^	5 ± 7	0.60 ± 0.21^a^	0.41 ± 0.22	0.42 ± 0.21^a^	0.16 ± 0.23
Ear height (cm)	10 ± 5^a^	4 ± 4	11 ± 4^a^	^c^	0.43 ± 0.21^a^	0.20 ± 0.23	0.55 ± 0.21^a^	^c^
Root lodging (%)	0	0	0	0	0	0	0	0
Stalk lodging (%)	0.113 ± 0.079	0.080 ± 0.056	^c^	0.152 ± 0.082	0.32 ± 0.22	0.31 ± 0.22	^c^	0.40 ± 0.22
Husk cover^d^	0.007 ± 0.010	0.005 ± 0.008	0.021 ± 0.013	0.013 ± 0.012	0.16 ± 0.23	0.16 ± 0.23	0.34 ± 0.22	0.24 ± 0.23
Plant aspect^e^	0.037 ± 0.018^a^	0.010 ± 0.014	0.023 ± 0.016	0.013 ± 0.013	0.42 ± 0.21^a^	0.17 ± 0.23	0.32 ± 0.22	0.21 ± 0.23
Ear aspect^f^	0.009 ± 0.013	0.028 ± 0.016	0.008 ± 0.021	0.009 ± 0.016	0.16 ± 0.23	0.38 ± 0.22	0.09 ± 0.24	0.13 ± 0.23
Ear rot (%)	0.030 ± 0.049	0.032 ± 0.043	0.116 ± 0.079	0.045 ± 0.041	0.14 ± 0.23	0.17 ± 0.23	0.32 ± 0.22	0.25 ± 0.22
Ears per plant	^c^	0.0006 ± 0.0007	0.0011 ± 0.0007	0.0019 ± 0.0009^a^	^c^	0.20 ± 0.23	0.36 ± 0.22	0.47 ± 0.21^a^
Stay green characteristic^g^	^c^	0.032 ± 0.031	0.005 ± 0.021	0.005 ± 0.026	^c^	0.23 ± 0.23	0.06 ± 0.24	0.04 ± 0.24

^a^, ^b^ = significantly different from zero at 0.05 and 0.01 levels of probability, respectively; ^c^ − negative variances treated as zero; ^d^Husk cover scored on a scale of 1–9, where 1 = husks tightly arranged and extended beyond the ear tip and 9 = ear tips exposed.; ^e^Plant aspect recorded on a scale of 1–9 based on plant type, where 1 = excellent and 9 = poor; ^f^Ear aspect rated on a scale of 1–9, where 1 = clean, uniform, large, and well-filled ears and 9 = ears with undesirable features; ^g^Stay green characteristic scored on a scale of 1–9, where 1 represented plants with almost all leaves green and 9 indicated plants with virtually all leaves dead

Under artificial *Striga* infestation, significant genetic variances were detected for grain yield, plant height, ear height and emerged *Striga* plants (8 WAP) in cycle C_0_, stalk lodging in C_1_, plant height and *Striga* damage (8 WAP) in C_2_ and days to anthesis and silking, plant height and ear height in cycle C_3_ (Table 4). Significant heritability estimates ranged from 0.43 for grain yield to 0.68 for plant height in C_0_ and 0.56 for plant height to 0.70 for days to anthesis in C_4_. Stalk lodging showed significant heritability estimate of 0.42 in C_1_ while plant height and *Striga* damage (8 WAP) had significant heritabilities of 0.54 and 0.58 in C_2_, respectively.

**Table 4:** Estimates of genetic variance (±SE) and broad-sense heritability (±SE), of measured traits of testcrosses involving early-maturing yellow S_1_ families derived from four cycles of selection and tester TZEI 23, evaluated under artificial *Striga* infestation at Mokwa and Abuja in Nigeria, 2014

Trait	Genetic variances				Broad-sense heritability			
	C_0_	C_1_	C_2_	C_3_	C_0_	C_1_	C_2_	C_3_
Grain yield (kg ha^− 1^)	71,631 ± 35488^a^	60,399 ± 56,601	80,359 ± 50,689	14,748 ± 41,440	0.43 ± 0.21^a^	0.24 ± 0.23	0.35 ± 0.22	0.08 ± 0.24
Days to anthesis	0.41 ± 0.27	0.10 ± 0.14	0.16 ± 0.14	0.59 ± 0.17^b^	0.33 ± 0.22	0.16 ± 0.23	0.27 ± 0.22	0.70 ± 0.20^b^
Days to silking	0.50 ± 0.32	0.13 ± 0.19	0.23 ± 0.16	0.72 ± 0.22^b^	0.34 ± 0.22	0.16 ± 0.23	0.32 ± 0.22	0.66 ± 0.20^b^
Anthesis-silking interval (days)	0.006 ± 0.012	0.010 ± 0.036	^c^	0.008 ± 0.014	0.12 ± 0.23	0.07 ± 0.24	^c^	0.12 ± .023
Plant height (cm)	34 ± 10^b^	13 ± 10	27 ± 10^a^	17 ± 6^a^	0.68 ± 0.20^b^	0.29 ± 0.22	0.54 ± 0.21^a^	0.56 ± 0.21^a^
Ear height (cm)	7.6 ± 2.98^a^	4.5 ± 4.62	4.2 ± 3.68	9.9 ± 3.13^b^	0.53 ± 0.21^a^	0.22 ± 0.23	0.26 ± 0.22	0.65 ± 0.20^b^
Root lodging (%)	0.028 ± 0.22	0.076 ± 0.13	^c^	0.167 ± 0.12	0.03 ± 0.24	0.14 ± 0.23	^c^	0.32 ± 0.22
Stalk lodging (%)	0.20 ± 0.19	0.44 ± 0.22^a^	0.20 ± 0.21	0.25 ± 0.18	0.24 ± 0.23	0.42 ± 0.21^a^	0.22 ± 0.23	0.30 ± 0.22
Husk cover ^d^	0.009 ± 0.012	0	0.014 ± 0.013	^c^	0.16 ± 0.23	0	0.25 ± 0.22	^c^
Plant aspect ^e^	0.003 ± 0.014	0.044 ± 0.025	^c^	0.021 ± 0.028	0.05 ± 0.24	0.38 ± 0.22	^c^	0.17 ± 0.23
Ear rot (%)	^c^	0.050 ± 0.071	0.073 ± 0.069	^c^	^c^	0.16 ± 0.23	0.24 ± 0.23	^c^
Ear aspect ^f^	0.0012 ± 0.0015	^c^	0.0010 ± 0.0010	^c^	0.18 ± 0.23	^c^	0.22 ± 0.23	^c^
*Striga* damage (8 WAP^g^)	0.012 ± 0.014	0.044 ± 0.024	0.073 ± 0.026^a^	0.024 ± 0.024	0.19 ± 0.23	0.40 ± 0.22	0.58 ± 0.21^a^	0.23 ± 0.23
*Striga* damage (10 WAP)	0.004 ± 0.008	0.012 ± 0.014	0.017 ± 0.017	0.012 ± 0.017	0.13 ± 0.23	0.20 ± 0.23	0.23 ± 0.23	0.16 ± 0.23
Emerged *Striga* plants (8 WAP)	0.062 ± 0.029^a^	^c^	^c^	^c^	0.45 ± 0.21^a^	^c^	^c^	^c^
Emerged *Striga* plants (10 WAP)	0.057 ± 0.032	0.028 ± 0.034	^c^	0.008 ± 0.025	0.39 ± 0.22	0.19 ± 0.23	^c^	0.08 ± 0.24

^a^, ^b^ = significantly different from zero at 0.05 and 0.01 levels of probability, respectively; ^c^ − negative variances treated as zero; ^d^Husk cover scored on a scale of 1–9, where 1 = husks tightly arranged and extended beyond the ear tip and 9 = ear tips exposed.; ^e^Plant aspect recorded on a scale of 1–9 based on plant type, where 1 = excellent and 9 = poor; ^f^Ear aspect rated on a scale of 1–9, where 1 = clean, uniform, large, and well-filled ears and 9 = ears with undesirable features; ^g^WAP = weeks after planting

Under optimal environments, significant genetic variances were observed for grain yield and plant height in C_0_, plant aspect in C_1_ and plant and ear heights in C_2_ (Table 5). Significant heritabilities of 0.44 and 0.60 were obtained for grain yield and plant height in C_0_. Plant aspect had significant heritability of 0.44 in C_2_ while significant heritabilities of 0.50 and 0.40 were observed for plant and ear heights in C_2_. It was striking that no trait had significant genetic variances and heritability estimates in cycle C_3_.

**Table 5:** Estimates of genetic variance (±SE) and broad-sense heritability (±SE), of measured traits of testcrosses involving early-maturing yellow S_1_ families derived from four cycles of selection and tester TZEI 23, evaluated optimal environments at Ikenne, Mokwa and Bagauda, 2014

Trait	Genetic variances				Broad-sense heritability			
	C_0_	C_1_	C_2_	C_3_	C_0_	C_1_	C_2_	C_3_
Grain yield (kg ha^− 1^)	115,786 ± 56807^a^	50,595 ± 54,256	70,055 ± 50,945	43,198 ± 38,297	0.44 ± 0.21^a^	0.21 ± 0.23	0.30 ± 0.22	0.25 ± 0.22
Days to anthesis	0.07 ± 0.09	0.11 ± 0.08	0.21 ± 0.14	0.15 ± 0.08	0.17 ± 0.23	0.31 ± 0.22	0.34 ± 0.22	0.39 ± 0.22
Days to silking	0.03 ± 0.10	0.21 ± 0.12	0.27 ± 0.15	0.16 ± 0.09	0.06 ± 0.24	0.36 ± 0.22	0.40 ± 0.22	0.36 ± 0.22
Anthesis-silking interval (days)	^c^	0.019 ± 0.05	0.015 ± 0.04	0.026 ± 0.04	^c^	0.09 ± 0.24	0.09 ± 0.24	0.14 ± 0.23
Plant height (cm)	22.440 ± 7.7^a^	0.019 ± 4.5	12.457 ± 5.2^a^	3.977 ± 3.3	0.60 ± 0.21^a^	0	0.50 ± 0.21^a^	0.27 ± 0.22
Ear height (cm)	4.163 ± 2.9	3.361 ± 3.0	7.757 ± 3.3^a^	5.951 ± 3.3	0.32 ± 0.22	0.25 ± 0.22	0.49 ± 0.21^a^	0.39 ± 0.22
Root lodging (%)	0	0	0	0	0	0	0	0
Stalk lodging (%)	0.04 ± 0.1	^c^	^c^	0.01 ± 0.1	0.13 ± 0.23	^c^	^c^	0.03 ± 0.24
Husk cover^d^	^c^	0.003 ± 0.003	^c^	^c^	^c^	0.19 ± 0.23	^c^	^c^
Plant aspect ^e^	0.004 ± 0.003	0.009 ± 0.004^a^	0.003 ± 0.003	0.005 ± 0.003	0.29 ± 0.22	0.44 ± 0.21^a^	0.21 ± 0.23	0.37 ± 0.22
Ear aspect^f^	0.0007 ± 0.007	0.0123 ± 0.011	0.0079 ± 0.010	0.0128 ± 0.009	0.02 ± 0.24	0.26 ± 0.22	0.19 ± 0.23	0.31 ± 0.22
Ear rot (%)	0.02 ± 0.08	0.02 ± 0.05	0.03 ± 0.07	0.01 ± 0.05	0.05 ± 0.24	0.07 ± 0.24	0.09 ± 0.24	0.06 ± 0.24
Ears per plant	0.0013 ± 0.001	^c^	0.0010 ± 0.001	0.0001 ± 0.001	0.26 ± 0.22	^c^	0.27 ± 0.22	0.06 ± 0.24

^a^, ^b^ = significantly different from zero at 0.05 and 0.01 levels of probability, respectively; ^c^ − negative variances treated as zero; ^d^Husk cover scored on a scale of 1–9, where 1 = husks tightly arranged and extended beyond the ear tip and 9 = ear tips exposed.; ^e^Plant aspect recorded on a scale of 1–9 based on plant type, where 1 = excellent and 9 = poor; ^f^Ear aspect rated on a scale of 1–9, where 1 = clean, uniform, large, and well-filled ears and 9 = ears with undesirable features

### Interrelationships among traits

**Fig. 2:**
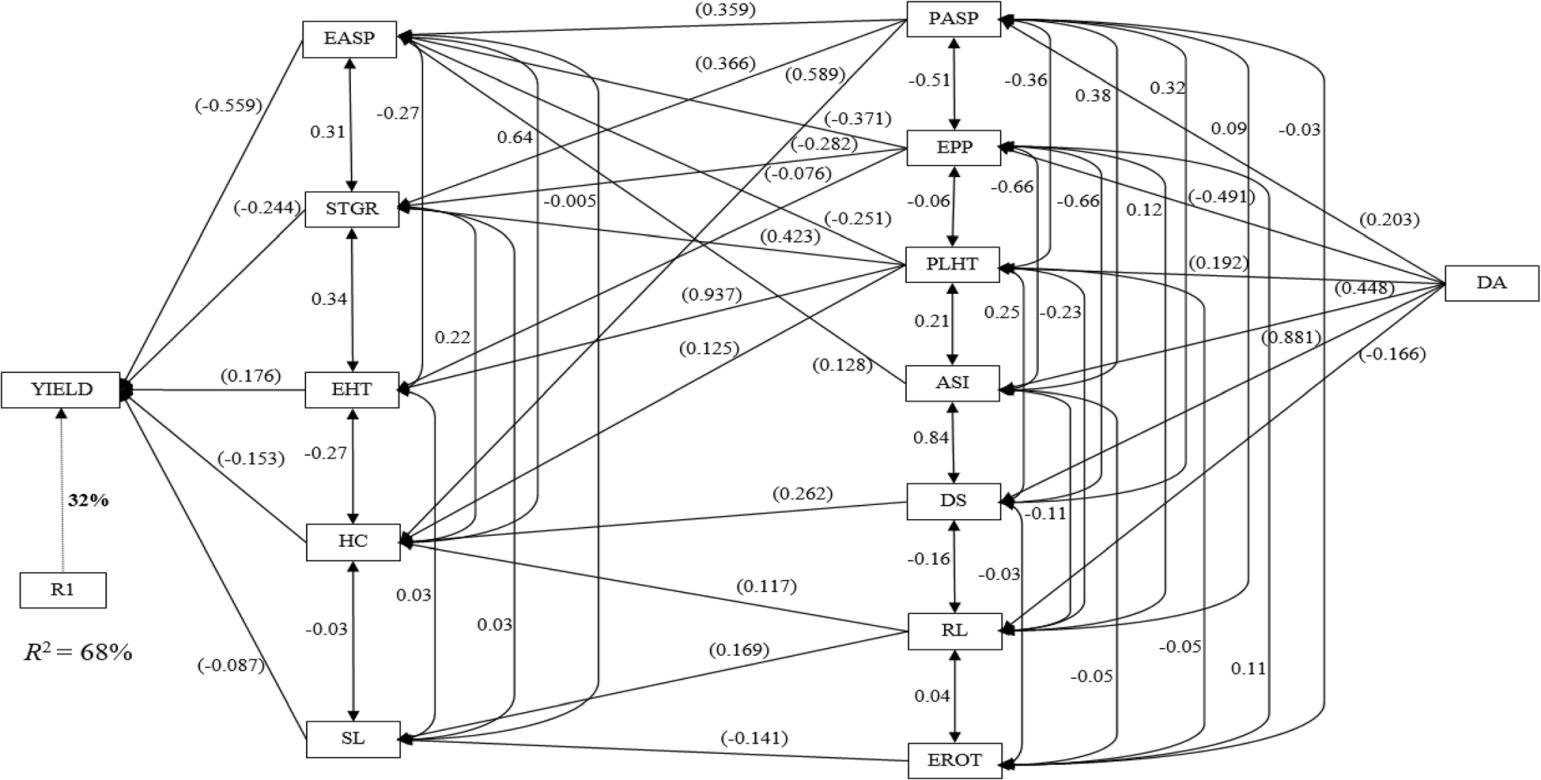
Path analysis model diagram showing causal relationships of measured traits of testcrosses involving early-maturing yellow S_1_ families derived from four cycles of selection and tester TZEI 23, evaluated across drought stress at Ikenne during the 2014/2015 and 2016/2017 dry seasons and Kadawa during the 2015 dry season in Nigeria. Bold values are residual effect; values in parenthesis are direct path coefficient and other values are correlation coefficients. R1 is the residual effects; ASI, anthesis–silking interval; DA, days to 50% anthesis; DS, days to 50% silking; EASP, ear aspect; EHT, ear height; EPP, ears per plant; EROT, ear rot; HC, husk cover; PASP, plant aspect; PLHT, plant height; STGR, stay green characteristic; RL, root lodging; SL, stalk lodging, and YIELD, grain yield

Under drought stress, the step-wise regression analyses identified ear aspect, stay green character, ear height, husk cover and stalk lodging as first order traits (primary contributors to grain yield), accounting for about 68% of the variation observed in grain yield (Fig. 2). Ear aspect had the highest direct effect (− 0.559) while stalk lodging had the least (− 0.087). Second order traits included plant aspect, ears per plant, plant height, anthesis-silking interval, days to silking, root lodging and ear rot. Plant height contributed indirectly to grain yield through four first order traits while plant aspect and ears per plant contributed indirectly to grain yield through three of the first order traits. Root lodging was an indirect contributor to grain yield through husk cover and stalk lodging, two first order traits. However, days to silking, anthesis-silking interval and ear rot contributed indirectly to grain yield through one first order trait. Days to anthesis was the only trait categorized as third order trait under drought.

**Fig. 3:**
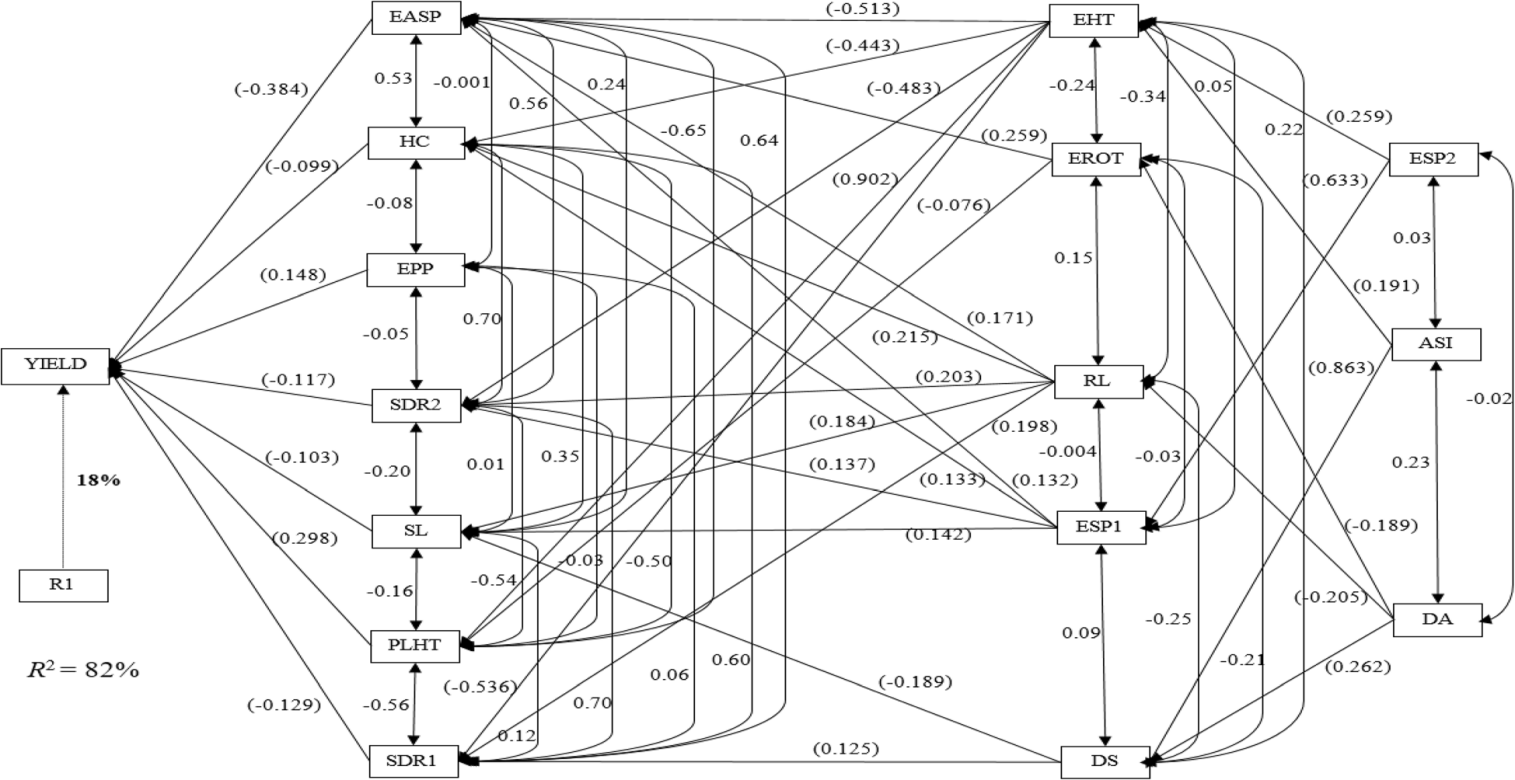
Path analysis model diagram showing causal relationships of measured traits of early yellow testcrosses evaluated across artificial *Striga* infestation at Mokwa and Abuja, 2014. Bold values are residual effect; values in parenthesis are direct path coefficient and other values are correlation coefficients. R1 is the residual effects; ASI, anthesis–silking interval; DA, days to 50% anthesis; DS, days to 50% silking; EASP, ear aspect; EHT, ear height; EPP, ears per plant; EROT, ear rot; ESP 1 and ESP 2, emerged *Striga* plants (8 and 10 WAP); HC, husk cover; PLHT, plant height; RL, root lodging; SDR 1 and SDR 2, *Striga* damage (8 and 10 WAP); SL, stalk lodging and YIELD, grain yield

**Fig. 4:**
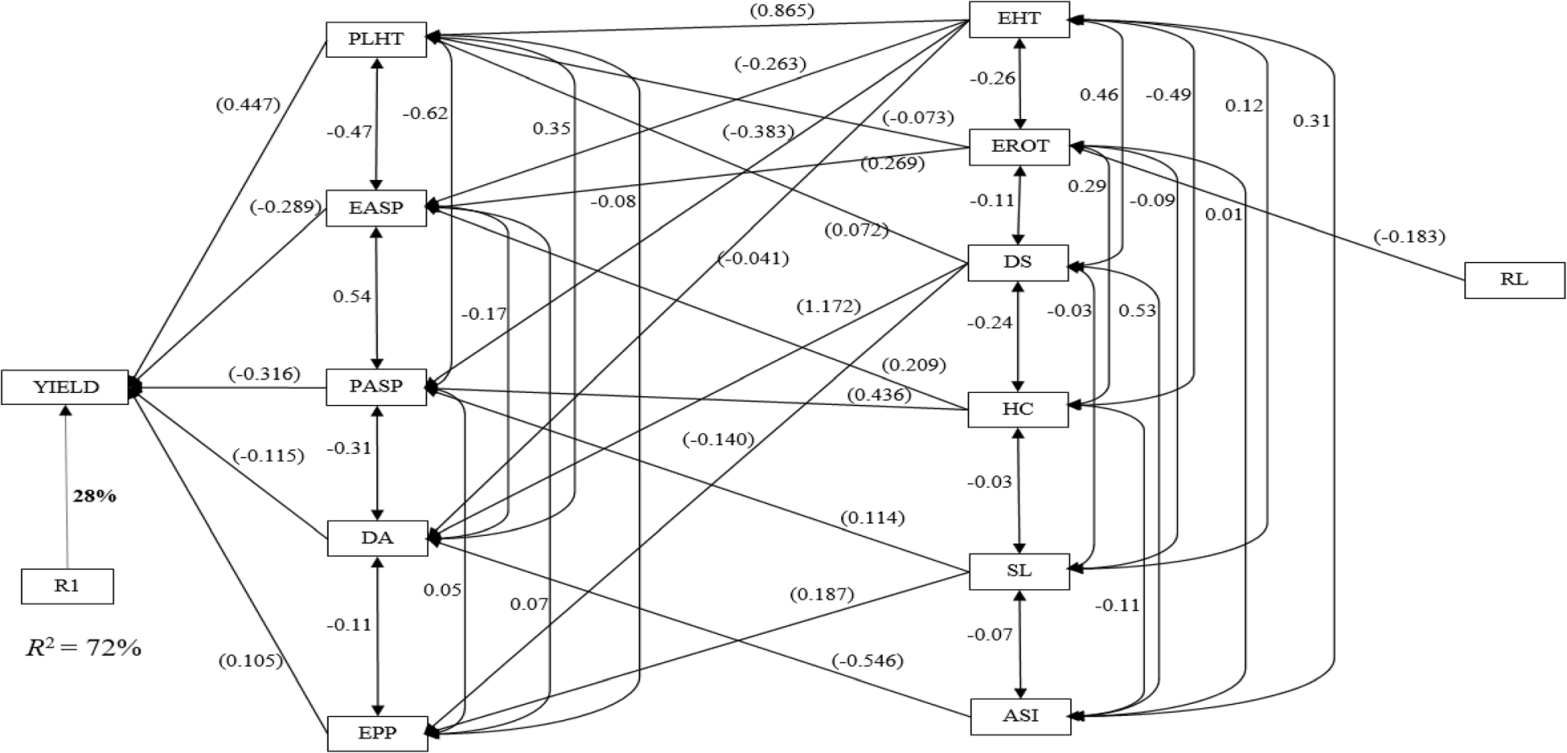
Path analysis model diagram showing causal relationships of measured traits of early yellow testcrosses evaluated across optimal growing environments at Ikenne, Mokwa and Bagauda during the 2014 growing season. Bold values are residual effect; values in parenthesis are direct path coefficient and other values are correlation coefficients. R1 is the residual effects; ASI, anthesis–silking interval; DA, days to 50% anthesis; DS, days to 50% silking; EASP, ear aspect; EHT, ear height; EPP, ears per plant; EROT, ear rot; HC, husk cover; PASP, plant aspect; PLHT, plant height; RL, root lodging; SL, stalk lodging, and YIELD, grain yield

Under *Striga* infestation, ear aspect, husk cover, ears per plant, stalk lodging, plant height and *Striga* damage (8 and 10 WAP) were the primary contributors to grain yield. These traits accounted for 82% of the variation in grain yield (Fig. 3). Out of the first order traits, ear aspect had the highest direct effect while husk cover had the least. The second order traits consisted of days to silking, ear height, ear rot, root lodging and emerged *Striga* plants (8 WAP) while the third order traits comprised days to anthesis, anthesis-silking interval and emerged *Striga* plants. Days to anthesis, plant height, plant and ear aspect and ears per plant were categorized as first order traits, which accounted for 72% of the variation in grain yield under optimal environments (Fig. 4). Plant height had the greatest contribution to grain yield with a direct effect of 0.447 while ears per plant was the least contributor with a direct effect of 0.105. Days to silking, anthesis-silking interval, ear height, husk cover, stalk lodging and ear rot were identified as second order traits. Root lodging was the only trait classified into the third order group under optimal environments.

## Discussion

### Analyses of variance and genotype x environment interaction

The significant environment mean square observed for grain yield and other assayed traits under drought, *Striga-*infested and optimal conditions in the present study indicated that the environments showed uniqueness in discriminating among the genotypes under each research condition [20]. This could be attributed to the varying environmental factors including amount of incoming solar radiation, temperature, soil type, rainfall pattern and disease pressure at the research environments [4]. The significant cycle effects observed for grain yield and most other agronomic traits measured under drought, *Striga-*infested and optimal growing environments suggested that there were differential responses in the different cycles which could facilitate selection under the contrasting research conditions. Under drought, the observed significant entry-within-cycle effects for grain yield and other measured agronomic traits except for anthesis-silking interval, root lodging, ears per plant and ear aspect implied that the testcrosses within each cycle varied in the expression of the traits. The testcrosses within each selection cycle also showed variability for the expression of traits including days to anthesis, days to silking, plant height, stalk lodging and *Striga* damage (8 WAP) under *Striga* infestation and grain yield, days to anthesis, days to silking, anthesis-silking interval, plant height, ear height, stalk lodging, plant aspect and ear aspect under optimal environments. The significant env x cycle interaction effects detected for all agronomic traits assayed under drought, *Striga*-infested and optimal environments with the exception of ear height, stalk lodging, husk cover, *Striga* damage (8 WAP), number of emerged *Striga* plants (8 WAP) and ears per plant under *Striga-*infested environments and days to anthesis, root lodging and plant height under optimal environments suggested that the traits in the selection cycles were not expressed in a consistent manner from one environment to the other. In contrast, non-significant entry-within-cycle x env interaction effect observed for all traits assayed under drought and *Striga* as well as traits other than days to anthesis, days to silking, stalk lodging and anthesis-silking interval under optimal environments indicated that the S_1_ lines within cycles were consistent in the expression of most agronomic traits measured under each of the research conditions.

### Yield gains and changes in other agronomic traits

A primary aim of this study was to determine yield gains following genomic selection, involving one PS cycle followed by two selection cycles based on molecular markers for *Striga* resistance and drought tolerance. The observed yield gains of 498 and 522 kg ha^− 1^ cycle^− 1^ which corresponded to 16.9 and 12.6% gain cycle^− 1^ under *Striga*-infested and optimal environments implied that genomic selection was effective for improvement of the population for grain yield under *Striga-*infested environments and resulted in concomitant increase in grain yield of the population under optimal environments. Since there are presently no documented reports on grain yield gains through genomic selection under *Striga-*infested conditions, the results of this study were compared with those obtained from S_1_ family selection under artificial *Striga* infestation. The gain of 498 kg ha^− 1^ cycle^− 1^ obtained for yield under *Striga* infestation is greater than the 52 kg ha^− 1^ realized gain documented by Badu-Apraku et al. [21] following four S_1_ family selection cycles for improved grain yield as well as resistance to *Striga hermonthica* in TZE-Y Pop DT STR, an early-maturing maize population. Similarly, the gain of 522 kg ha^− 1^ cycle^− 1^ obtained for grain yield under optimal environments was higher than that of Badu-Apraku et al. [21] who reported yield gains of 130 kg ha^− 1^ cycle^− 1^ under optimal conditions after subjecting an early maturing maize population to four S_1_ family selection cycles for improvement of grain yield along with *Striga* resistance. The 162 kg ha^− 1^ cycle^− 1^ grain yield gains obtained by Edmeades et al. [22] in two maize populations of early maturity, Pool 16 Sequia (after 1 cycle) and Pool 18 Sequia (after 3 cycles) under well-watered conditions is also less than the yield gain obtained under optimal environments in this study. The observed differences between our results and the findings of other authors could be due to the differences in the germplasm and/or the selection procedures. Our findings clearly demonstrated the superiority of genomic selection over conventional phenotypic selection methods for grain yield improvement under *Striga* infestation and by extension, under optimal environments. However, the lack of yield gain under drought environments implied that genomic selection was ineffective for yield improvement under this stress factor probably due to low marker density, more minor gene loci, low heritability or reliability of phenotyping. Our findings in the present study is contrary to that of Beyene et al. [23] who obtained an average of 86 kg ha^− 1^ yield gain in eight bi-parental maize populations following genomic selection under managed drought environments. A plausible explanation for the differences between our findings and the results obtained by Beyene et al. [23] could be the dissimilarity of the genetic materials and methodology used or the possibility of trade-offs while improving the bi-parental population concurrently for *Striga* resistance and tolerance to drought. In addition, Bankole et al. [24] evaluated S_1_-testcrosses developed from three selection cycles (C_0_, C_1_, C_2_) of a late-intermediate bi-parental population, following marker-assisted recurrent selection. The authors reported higher grain yield from S_1_-testcrosses involving the C_2_ population compared with those generated from the C_0_ of the population, corresponding to a grain yield gain of 7% per cycle. In contrast, Abdulmalik et al. [25] detected no significant gain in yield following four selection cycles involving molecular markers in a late-intermediate bi-parental maize population under drought. The authors explained that the findings could have been confounded by the combined effects of drought and army worms during evaluation in the field. These contrasting results indicated that GS just like any other selection method could be efficient for improvement of a genetic material or breeding population depending on the genetic variability, breeding objectives and the methodology employed.

Furthermore, the phenotypic selection index used in the present study integrated increased values of grain yield and higher ears per plant along with decreased values of anthesis-silking interval, plant aspect, ear aspect, *Striga* damage (8 and 10 WAP), number of emerged *Striga* plants (8 and 10 WAP) and stay green characteristic, which in combination with the genotypic data of the training population were used to compute the GEBV [26–28]. However, the association of yield gains under *Striga* with increase in plant height and ear height as well as improvement in resistance to root lodging, ear aspect, husk cover and *Striga* tolerance (i.e. decrease in *Striga* damage scored at 8 and 10 WAP) implied that genomic selection effectively improved grain yield and resistance to *Striga* and ear aspect, but could not keep the plant and ear heights constant. This is also true for the yield gains obtained under optimal environments which was accompanied by increased plant and ear heights along with improvement of husk cover and ear rot resistance. Under drought, however, genomic selection was ineffective in improving grain yield and resulted in delayed flowering, poor anthesis-silking synchrony during flowering and increased ear height, which were contrary to the expectations.

### Genetic variances and heritability estimates

For a recurrent selection program to be considered as effective and successful it should simultaneously increase the average performance of individuals in the population and at the same time maintain or increase the genetic variability of traits in the population [4, 15, 21, 29]. One of the aims of this study was therefore to examine critically the estimates of genetic variances and heritability of traits during GS under *Striga* infestation, managed drought and optimal environments. The non-significant genetic variances and heritabilities of most agronomic traits assayed in all the selection cycles suggested low gene frequencies of the traits in the population [4]. In the most advanced selection cycle, C_3_, the lack of significant genetic variances and heritabilities estimated for grain yield and most measured agronomic traits except ears per plant under drought indicated that the residual variability for most traits would not suffice for progress from further GS in the population. Similar trends were observed in C_3_ for all measured traits under optimal environments and most measured traits under *Striga*-infested environments except for days to anthesis, days to silking, plant and ear heights. Our findings are not surprising since the population was improved for *Striga* resistance and drought tolerance using the GS that originated from only two inbred lines and therefore had a narrow genetic base to begin with. Therefore, there is a need to introgress novel sources of favourable *Striga* resistance and drought tolerance alleles into the population to ensure further progress from GS for yield improvement, *Striga* resistance, and drought tolerance [29].

### Interrelationships among traits

Index selection has long replaced selection solely for grain yield under stress conditions. This is because grain yield usually has low heritability under stress factors while some reliable secondary traits maintain high heritabilities under these stress conditions [30, 31]. In the present study, step-wise regression and sequential path analyses were used to investigate the cause and effect relationships involving grain yield and other agronomic traits assayed under the different research conditions. The sequential path analysis is a unique approach that identifies traits making direct contributions to grain yield and categorises them as first order traits, followed by traits with indirect contributions to grain yield through traits of the first order, which are categorized as second order traits and so on [32, 33]. This approach facilitates the classification of secondary traits in declining order of their relative contributions to the observed variation in grain yield. Identification of ear aspect, stay green character, ear height, husk cover and stalk lodging as traits of the first order under drought stress was an indication that these secondary traits were important for yield improvement under the stress factor. Contrary to the results of this study, Talabi et al. [33] identified anthesis-silking interval, stay green characteristic, ear aspect, plant aspect, and ears per plant as the most important traits accounting for the variation in grain yield. Under *Striga*-infested environments, husk cover, ear aspect, ears per plant, stalk lodging, plant height and *Striga* damage (8 and 10 WAP) were categorized as traits of the first order, playing key roles in explaining the variation observed in grain yield. This result agrees partially with the results of Badu-Apraku et al. [34] where ear aspect and ears per plant were identified as the only direct contributors to grain yield. The identification of days to anthesis, plant height, plant aspect, ear aspect and ears per plant as first order traits under optimal environments implied that the traits served as key players for improvement of grain yield under optimal environments even though GS placed emphasis on improvement in grain yield under *Striga* infestation and water deficit environments. Badu-Apraku et al. [35] also identified plant and ear aspects as the first order traits responsible for about 94% of the variation in grain yield under optimal or high-N environments. One important information from the present study and those of previous researchers was the identification of ear aspect as a primary contributor to grain yield under *Striga*-infested, drought, low-N and optimal environments [32, 33, 35]. This indicated that ear aspect could serve as an indirect selection criterion for simultaneous improvement of grain yield under *Striga*, low-N, drought and optimal environments. However, scoring for this trait is usually done by trained plant breeders. Promotion and wide adoption of ear aspect by plant breeders would improve the efficiency of breeding maize for tolerance to multiple stresses in the sub-region.

## Conclusions

This study has led to the conclusion that genomic selection was effective for grain yield improvement under *Striga* infestation and resulted in concomitant increase in yield performance under optimal environments. However, under drought environments, genomic selection was ineffective for improvement of grain yield. The yield gain under *Striga* was associated with increase in plant height and ear height as well as improvement in resistance to root lodging, ear aspect, husk cover, and *Striga* tolerance while gain in grain yield under optimal environments was accompanied by increase in plant height and ear height along with improvement of husk cover and ear rot resistance. The result of genomic selection was delayed flowering, poor anthesis-silking synchrony during flowering and increased ear height under drought environments. In addition, most traits lacked genetic variability in all the selection cycles particularly the cycle C_3_, necessitating introgression of novel and beneficial drought tolerance and *Striga* resistance alleles into the population to ensure progress from further GS. Ear aspect is a key trait that could serve as indirect selection criterion for simultaneous improvement of grain yield under *Striga*, drought and optimal environments.

## Methods

### Development of the genetic material

In 2007, two yellow maize inbred lines of early maturity, TZEI 17 (*Striga* resistant) and TZEI 11 (drought tolerant) were selected using the available field data, and inter-crossed to form the bi-parental population, TZEI 17 x TZEI 11. Seeds from the parental inbred lines along with leaf samples harvested from the bi-parental cross at the 3–5 leaf stage were genotyped to confirm the presence of parental type alleles in the bi-parental cross. A total of 108 SNPs were initially generated, after screening of over a thousand KASP assays which were developed by LGC Genomics (United Kingdom) following the conversion of 1536 Illumina Golden Gate Array [36]. However, six SNPs were bad and uncallable; thus, they were not used for the GS. The SNP markers used for GS were: (i) uniform and homozygous in the parental lines, (ii) polymorphic in the parental lines and (iii) heterozygous in the bi-parental population. A total of 15, 20, 17, 5, 14, 12, 7, 7, 2 and 3 SNPs were distributed on chromosomes 1 to 10, respectively (Table 6). Although the 102 SNPs used for GS in the present study fell below the required minimum of approximately 200 SNPs recommended by Zhang et al. [37] for GS in bi-parental populations, they were the only markers that showed polymorphism for the parent type alleles in the bi-parental population and were therefore used for the GS. The bi-parental population, TZEI 17 x TZEI 11 was subjected to two successive cycles of self-pollination to generate S_2_ lines (F_3_ progenies) used as cycle C_0_ (base population) for GS. A total of 382 S_2_ lines developed from the bi-parental population TZEI 17 x TZEI 11, were inter-mated with TZEI 23, a standard inbred tester of opposing heterotic group, during the dry season of 2009/2010 in Ibadan. These testcrosses were tested at Mokwa and Abuja under artificial *Striga* infestation and at Ikenne under optimal growing conditions all in 2010. The testcrosses were equally evaluated under random drought at Bagauda in Nigeria and Chiredzi in Zimbabwe, 2010 and under managed drought at Ikenne during the dry season of 2010/2011. Leaf samples of the S_2_ lines of the bi-parental population were harvested in the field, for genomic DNA extraction and genotyping using the full complement of the 102 SNPs.

**Table 6:** Distribution of 102 SNPs on the 10 maize chromosomes in this study

Chromosome number	Associated SNPs	Total number of SNPs per chromosome number
1	PHM14475_7, PZA00343_31, PZA03578_1, PHM4997_11, PZA00887_1, PZA03457_1, PZB00648_5, PZA02195_1, PZA02284_1, PHM1968_22, PZA01588_1, PZA02278_1, PZB00114_1, d8_2 and PHM9418_11.	15
2	PZA00527_10, PZA01991_3, PHM1511_14, PZA00108_4, PHM482_23, PHM7953_11, PZA02133_10, PZB00183_4, PZA03228_4, PHM5060_12, PZA02417_2, PZA03529_1, PHM793_25, PZA02890_4, PHM6111_5, PHM4880_179, PHM3626_3, PZD00022_5, PZA02453_1 and PZA02418_2.	20
3	PZA02296_1, PZA02402_1, PZA03458_1, PHM12859_7, PHM15475_27, PZA00413_20, PHM3352_21, PZB01109_1, PZA03391_1, PZA01765_1, PZD00038_2, PZA00109_4, PHM5502_31, PZA03647_1, PZA03070_9, PZA02699_1 and PZA02616_1	17
4	PZA00529_4, PZA02289_2, PHM14618_11, PZA01658_1 and PHM3155_14	5
5	PZA00517_7, PZA00996_1, PZA01887_1, PZA02029_21, PZA03167_5, PZA03452_6, PZA00395_2, PZA00352_23, PZA03324_1, PHM2524_4, PHM7908_20, PZB00765_1, PHM7908_25 and PHM4165_14	14
6	PZA00266_7, PZA00910_1, PZA01462_1, PZA03027_12, PZA03047_12, PZB01009_2, PHM4904_16, PHM5529_7, PZB01222_1, PZA02678_1, PHM2551_31 and PZA00355_2	12
7	PZA00256_27, PZA03645_1, PZA02373_1, PHM4353_31, PZA03363_1, PZA03344_2 and PZA01909_2	7
8	PZA01257_1, PZA03182_5, PZA00717_15, PHM2749_10, PHM2350_17, PZA01741_1 and PZA01186_1	7
9	PZA00466_1 and PHM1911_173	2
10	PZA02221_20, PZA02663_1 and PHM3312_23	3

In order to advance the population to Cycle 1 of the selection program, a phenotypic selection index (PSI) comprising grain yield, anthesis-silking interval, plant aspect, ear aspect, ears per plant, *Striga* damage at 8 and 10 WAP, number of emerged *Striga* plants at 8 and 10 WAP and stay green characteristic was computed using the results of the multi-location trials. The Best Linear Unbiased Prediction (BLUP) [26] was used to calculate the marker effect of the 382 F_3_ lines while a genomic relationship matrix following the methods of Habier et al. [27] and VanRaden [28], was used to compute the genomic estimated breeding values (GEBV). Based on the presence or absence of parental type alleles, scores were assigned to each of the F_3_ lines while the marker score of each line was used to multiply the BLUP values per marker of the F_3_ lines to obtain the GEBVs. The PSI was used to select the top 10% F_3_ lines involved in the testcrosses developed from (TZEI 17 x TZEI 11) F_3_ and TZEI 23 (inbred tester), which were inter-mated to form the (TZEI 17 x TZEI 11) C_1_ in 2011, using the balanced composite approach. The C_1_ plants were genotyped with the full complement of SNPs used originally for the genotyping of the C_0_ population. Based on the SNPs data and PSI, the GEBV was computed for each individual plant in C_1_. The top 10% of the lines in C_1_ were selected using the GEBV and recombined following the balanced composite approach to constitute (TZEI 17 x TZEI 11) C_2_ in 2012. Genotyping was done for C_2_ plants and the protocol used for recombination in C_1_ was repeated in C_2_ to develop (TZEI 17 x TZEI 2) C_3_ during the dry season of 2012/2013 in the breeding nursery of IITA-Ibadan.

### Generation of testcross progenies

Fifty S_1_ families each were extracted from cycles C_0_, C_1_, C_2_ and C_3_ of TZE 17 x TZEI 11 F_3_ population during the 2013 growing season in Ibadan. The resulting 200 S_1_ lines were crossed to a tester of opposing heterotic group, TZEI 23 to generate testcrosses during the 2013/2014 dry season in the IITA-Ibadan breeding nursery. The 200 testcrosses were tested under managed drought, artificial *Striga* infestation and optimal growing environments in Nigeria, from 2014 to 2017.

### Field evaluation of testcross progenies

We conducted three field experiments between 2014 and 2017. The first experiment involved the testing of the 200 testcrosses under managed drought stress at Ikenne (6°53′N, 3°42′E, 60 m altitude, 1200 mm yearly rainfall), during the dry seasons of 2014/2015 and 2016/2017. These testcrosses were also evaluated under combined drought and heat stress at Kadawa (11°45′N, 8°45′E, 468.5 m altitude, 884 mm yearly rainfall) during the dry season of 2015. The drought trials were established at Ikenne during the dry season in November and supplied with 17 mm of water on weekly basis, with the aid of a sprinkler irrigation system. Managed drought stress was imposed at 28 days after planting, when supply of irrigation water was discontinued such that the plants had to depend on residual moisture in the soil for growth, flowering and grain-filling till physiological maturity. The experimental fields in Ikenne sub-station were fairly uniform and flat, with high water-retention capacity, and the soil type is eutric nitrisol [38]. The combined drought and heat trial was established at Kadawa in February, during the dry season. Water was supplied to the trial twice a week for the first 28 days, using furrow irrigation system. At 28 days after planting, irrigation was discontinued and consequently, the plants were subjected to severe combined heat and drought stress for three consecutive weeks in April, with day temperatures varying from 35 to 39 °C and night temperature ranging between 22 and 27 °C. Subsequently, supply of irrigation water continued once a week during grain filling till the crop attained harvest maturity (with day temperature range of 33 to 40 °C and night temperature range of 24 to 28 °C). The soil type at Kadawa is Cambisol [38]. Basal fertilization of the managed drought experiment at Ikenne and the combined heat and drought trial at Kadawa was carried out using 60 kg each of N, P_2_O_5_ and K_2_O ha^− 1^ at planting, while topdressing with an additional 60 kg of N ha^− 1^ was done at 4 WAP.

In the second experiment, the 200 testcrosses were tested under artificial infestation of *Striga* at Abuja (9^о^16’N, 7^о^20’E, altitude 300 m, 1500 mm yearly rainfall) and Mokwa (9°18′N, 5° 4′E, altitude 457 m, 1100 mm yearly rainfall) in the Southern Guinea Savanna of Nigeria in 2014. The fields were injected with ethylene gas at about 10 days before planting, to stimulate suicidal germination of residual *Striga* seeds in the soil. Artificial infestation of *Striga* was carried out following the method described by Kim [39] and Kim & Winslow [40]. *Striga* seeds sourced from neighbouring fields planted to sorghum were stored for about 6 months to break *Striga* seed dormancy and subsequently used for the infestation. Each hole in the *Striga* plot received about 500 germinable seeds of *Striga* mixed with fine sand in the ratio 1:99 following the procedure described by Kim [39]. Fertilizer application was delayed in the *Striga* experiment fields until about 25 days after planting (DAP) when 30 kg each of N, P and K ha^− 1^ was applied as NPK (15–15-15). The delay in fertilization and the reduced application rate were necessary precautions undertaken to enhance germination and emergence of *Striga* seeds as well as to facilitate the attachment of the *Striga* plants to the roots of the maize plant [39]. With the exception of *Striga* plants, all weeds were manually eliminated.

In the third experiment, the 200 testcrosses were evaluated under optimal growing environments i.e. environments free from *Striga* infestation and without limitations of water and nitrogen at Ikenne, Mokwa and Baguada (12°00′N, 8°22′E, 580 m altitude, 800 mm yearly rainfall) during the 2014 growing season. The trials received 60 kg N ha^− 1^, 60 kg P_2_O_5_ ha^− 1^ and 60 kg K_2_O ha^− 1^ at 2 weeks after planting (WAP) and were top-dressed with 60 kg/ha N at 4 WAP.

A 10 × 5 lattice design with two replications was used for the evaluation of the set of 50 testcrosses derived from the crosses involving 50 S_1_ lines of each selection cycle and the tester TZEI 23. Randomization was restricted within testcrosses of each selection cycle and the four cycles together constituted each trial in the present study. The experimental units were 3 m long single-row plots, with inter- and within-row spacings of 0.75 and 0.40 m, respectively. Three maize seeds were planted per hill but only two seedlings were retained per hill, following thinning at about 2 weeks after emergence. This gave a final population density of 66,666 plants ha^− 1^. For the *Striga* experiments, only pre-emergence herbicide was applied to control the weeds and was complemented with manual weeding. In all other experiments, weeds were controlled using Atrazine and Gramoxone as pre- and post-emergence herbicides, respectively at 5 L/ha each of Primextra and Paraquat.

### Data collection

In the managed drought, combined drought and heat, and optimal environments, data were recorded for days to 50% anthesis and silking (DA and DS), anthesis-silking interval (ASI), plant and ear heights (PLHT and EHT), root and stalk lodging (RL and SL), plant and ear aspects (PASP and EASP) and number of ears per plant (EPP). Details on how the traits were measured have been previously described by Badu-Apraku et al. [5]. Stay-green characteristic (STGR) was scored for the drought/combined drought and heat stress experiments at 70 DAP. For trials conducted under managed drought and combined heat and drought, grain yield (kg ha^− 1^) was computed from the weight of shelled kernels, adjusted to moisture content of 15%. In contrast, grain yield (kg ha^− 1^) for the optimal and *Striga* experiments, were estimated from field weight of ears per plot, assuming a shelling percentage of 80, adjusted to moisture content of 15%. Moisture content at harvest was recorded for representative shelled kernels per plot in all experiments using a moisture meter. The data recorded for *Striga* trials were essentially the same as those assayed under optimal experiment except that plant aspect was not scored. In addition, *Striga* damage [38] were scored at 8 and 10 WAP (SDR1 and SDR2) while the number of emerged *Striga* plants were also counted at 8 and 10 WAP (ESP1 and ESP2) in the *Striga*-infested plots. *Striga* damage was scored per plot on a scale of 1 to 9 where 1 = no damage, an indication of normal plant growth and high resistance, and 9 = total collapse or death of the maize plant, i.e., highly susceptible [39].

### Statistical analysis

The analysis of variance (ANOVA) was conducted across test environments for each experiment on plot mean basis for grain yield and other agronomic traits with PROC GLM in SAS [41], using a RANDOM statement with TEST option. Location-year combinations were treated as environments. In the combined ANOVA, genotypes were considered as fixed effects, while the test environments, replications, interaction of genotype × environment (G × E), and all other sources of variation were treated as random effects. The location–year combinations, replicate-within-environment, and block-within-replicate of each experiment were random factors, whereas entries were fixed effects. Mean separation was carried out using the LSD. Score and count data were subjected to natural logarithm transformation prior to ANOVA.

Means for grain yield and other agronomic traits of the testcrosses (dependent variables) were individually regressed on the selection cycles (independent variables) to obtain the coefficient of regression or gain cycle^− 1^ (b-value) under drought, *Striga*-infested and optimal environments. The b-value divided by the intercept and then multiplied by 100 provided an estimate of the relative percentage genetic gain per cycle.

As described by Hallauer et al. [15], estimate of broad-sense heritability was computed on a progeny-mean basis as follows:

$$ H=\frac{\sigma_{\mathrm{g}}^2}{\sigma_{\mathrm{g}}^2+\frac{\sigma_{\mathrm{g}\mathrm{e}}^2}{\mathrm{e}}+\raisebox{1ex}{${\sigma}^2$}\!\left/ \!\raisebox{-1ex}{$\mathrm{er}$}\right.} $$

where *r* = number of replications within environment; *e* = number of environments; σ_g_^2^ is the variance component due to genotypes; σ^2^_ge_ = variance component due to genotype × environment interactions; σ^2^ = the experimental error variance estimate. Standard errors associated with the genetic variances and heritabilities estimated were also computed as described by Hallauer et al. [15]. A variance or heritability estimate was considered significant if it had a value greater than two times the standard error while pair-wise comparison of estimates using corresponding standard errors was used to test for differences among the variances and heritability estimates of testcrosses from the different selection cycles.

The software SPSS version 17.0 [42] was used for step-wise regression analyses, to determine the causal relationships among traits of testcrosses in a sequential order under drought, *Striga* and optimal environments. Firstly, grain yield (primary trait) was regressed on all other agronomic traits to identify traits with direct significant (*P ≤* 0.05) contributions to grain yield which were categorized as traits in the first order. Secondly, each of the traits in the first order category was regressed on other measured agronomic traits not in the first order category, to identify those with indirect contributions to grain yield through the first-order traits, which were categorized as second-order traits. This procedure was repeated as applicable, to identify traits in the third, fourth orders. This approach facilitated the categorization of the predictor traits into first, second and third orders with minimized multicolinearity based on the respective contribution of the traits to the total observable variation in grain yield [33, 34]. The sequential path diagrams following the method of Mohammadi et al. [32] was used to depict the causal relationship among grain yield and other agronomic traits in a pictorial sequential order. The standardized *b* values obtained from the stepwise regression analyses provided the estimates of the path co-efficient [32–34]. The *t*-test (*P ≤* 0.05) was used to test the statistical significance of the path co-efficients. Thus, only traits showing significant path coefficients were captured in the model along with the percentage variation explained in the dependent variable(s).

## Abbreviations

ANOVA: Analysis of variance
ASI: Anthesis-silking interval
C_0_: Base population
C_1_: Cycle 1
C_2_: Cycle 2
C_3_: Cycle 3
DA: Days to 50% anthesis
DAP: Days after planting
DS: Days to 50% silking
EPP: Ear number per plant
GEBV: Genomic estimated breeding value
GS: Genomic selection
HUSK: Husk cover
IITA: International Institute of Tropical Agriculture
PHT: Plant height
SSA: sub-Saharan Africa
STGR: Stay green characteristic
WAP: Weeks after planting
